# Suppression Of Aberrant Activation Of NF-κB Pathway In Drug-resistant Leukemia Stem Cells Contributes To Parthenolide-potentiated Reversal Of Drug Resistance In Leukemia

**DOI:** 10.7150/jca.52641

**Published:** 2021-07-25

**Authors:** Juan Yi, Li Wang, Xiao-Yan Wang, Jing Sun, Xiao-Yang Yin, Jin-Xia Hou, Jing Chen, Bei Xie, Hu-Lai Wei

**Affiliations:** 1 School of Basic Medical Sciences, Lanzhou University; Key Laboratory of Preclinical Study for New Drugs of Gansu Province, 99 Dong Gang West Road, Lanzhou, 730000 Gansu, China; 2 Lanzhou University Second Hospital, 80 Cui Ying Men, Lin Xia Road, Lanzhou, 730000 Gansu, China; 3 Gansu Provincial Maternity and Childcare Hospital, 143 North Street, Qi Li He district, Lanzhou, 730050, Gansu, China; 4 State Key Laboratory of Bioactive Substance and Function of Natural Medicines, Institute of Materia Medica, Chinese Academy of Medical Sciences and Peking Union Medical College, Beijing 100050, P. R. China

**Keywords:** Drug-resistant leukemia stem cells, PTL, NF-κB signaling pathway, MDR, aberrant activation

## Abstract

Although many drugs that targeted the specific features of leukemia stem cells (LSCs) have substantial application in the clinical treatment of leukemia, the LSCs relapsed and caused drug-resistant leukemia. Therefore, it is necessary to identify the unique features of LSCs in relapsing and drug-resistant leukemia and also to explore the drugs that directed at these features. Our clinical data have indicated that relapsed patients with acute myeloid leukemia have more abundant proportion of LSCs with enhanced breast cancer resistance protein (BCRP) and P-glycoprotein (P-gp) expression when compared to the untreated patients. The results showed that compared with LSCs derived from sensitive K562 cells, LSCs from drug-resistant K562/ADM cells have much higher chemotherapeutic resistance, and so we termed these cells as “drug-resistant LSCs”. Subsequently, aberrant activation of NF-κB pathway in drug-resistant LSCs was further using gene chip analysis. Also, parthenolide (PTL), which is a specific NF-κB inhibitor, effectively eliminated drug-resistant LSCs and enhanced the sensitivity of K562/ADM cells to doxorubicin-induced apoptosis by down-regulating NF-κB pathway-mediated P-gp expression. These findings make the research area of LSCs more abundant and provide a potential therapeutic strategy for the treatment of refractory and relapsed leukemia.

## Introduction

Drug resistance, also named as multi-drug-resistance (MDR), has become a big challenge in the clinical treatment of leukemia, as it leads to recurrence of leukemia^1^, ^2^. The multiple mechanisms of MDR, including the over-expression of drug efflux, defective apoptotic mechanisms, and damaged DNA repair, have been explored previously^3^. Leukemia stem cells (LSCs) have been identified for the first time in 1990s, providing us a new horizon for exploring the mechanism of MDR. Previous studies have established that enriched CD34+CD38- leukemia cells assisted in sustaining serial transplantation and implicated in chemotherapy resistance^4^, ^5^. Furthermore, a higher frequency of relapse is associated with a higher proportion of LSCs^6^-^8^. Thus, LSCs represent a reservoir in driving chemotherapeutic resistance and relapse.

A growing body of evidence revealed that factors resulting in chemotherapy resistance of LSCs included the aberrant activation of critical signaling pathways^9^, ^10^, enhanced drug efflux^11^-^13^, defective apoptotic mechanisms^14^, ^15^, quiescent cell cycle^16^, the microenvironment^17^, dysregulation of metabolic pathways^18^, ^19^ and the epigenetic mechanisms^20^. According to these unique mechanisms, many researchers have focused on developing small molecules or drugs targeting the features in LSCs, such as the inhibitors that directed against some vital signaling pathways^21^, HDAC inhibitors^22^, G-CSF^16^, Bcl-2 inhibitors^23^, and inhibitors of drug efflux transporters^24^, in order to improve the chemosensitivity. Although a growing understanding on LSCs has provided opportunities on therapeutic targeting of these cells, and so they cannot be eliminated by employing these approaches, especially the LSCs derived from chemotherapeutic resistant and relapsed leukemia.

Our previous study revealed that LSCs derived from drug-resistant K562/ADM cells were not only significant in number but also demonstrated higher expression of ATP transporters (P-gp and BCRP) than LSCs in sensitive K562 cells. More interestingly, LSCs in K562/ADM cells demonstrated an increased resistance when compared with those in K562 cells^13^, ^25^. Furthermore, relapsed patients with acute myeloid leukemia (AML) had a more substantial proportion of LSCs along with enhanced breast cancer resistant protein (BCRP) and P-glycoprotein (P-gp) as compared with untreated patients^26^. According to the above cues, we set forth the existence of drug-resistant LSCs for the first time. Therefore, there is an urgent need to identify the characteristics of drug-resistant LSCs further and explore the natural compounds that target these cells to clear off drug resistance caused by drug-resistant LSCs.

Parthenolide (PTL) is the main active ingredient of feverfew, and belongs to the group of sesquiterpene lactones. Numerous studies have confirmed that PTL has vigorous antitumor activities and increased the sensitivity of other anti-cancer drugs^27^, ^28^. More importantly, PTL is the first efficacious natural drug directed against LSCs, which means that it can preferentially kill leukemia stem/progenitor cells, but with no effect on the non-cancer cells^29^.

Our previous experiments confirmed that PTL could induce apoptosis of K562 leukemia cells and its LSCs^25^. However, the effect of PTL on resistance of K562/ADM cells and drug-resistant LSCs has not been studied. Our study results revealed that in addition to overexpression of BCRP and P-gp, drug-resistant LSCs also demonstrated a higher expression of anti-apoptotic protein Bcl-2 in contrast to sensitive LSCs. Using RT2 Profiler PCR Array Human Signal Transduction Pathway Finder chip, dysregulation of NF-κB signaling pathway in drug-resistant LSCs was further confirmed, which is a distinct feature different from sensitive LSCs. Importantly, no difference in the effect of PTL on drug-resistant LSCs and sensitive LSCs has been found, implying that PTL could be a promising drug that reversed MDR by getting rid of drug-resistant LSCs. As for the mechanisms of cell death caused by PTL, our data showed that mitochondrial-dependent apoptosis, which was aroused by inhibiting the activity of NF-κB and increasing ROS levels, was still the main pathway. PTL also decreased the expression of P-gp in K562/ADM cells and drug-resistant LSCs by inhibiting the activity of NF-κB, thus elevating the concentration of doxorubicin in cells to promote apoptosis and ultimately reversing the resistance of cells to doxorubicin.

## Materials and methods

### Cells and cell culture

K562/ADM cells and K562 cells were maintained in RPMI1640 (Gibco, Thermo Fisher Scientific, USA) medium supplemented with 10% fetal bovine serum (FBS, GE Healthcare Life Sciences, USA), 1mM l-glutamine, 50Uml-1 penicillin, and 50Uml-1 streptomycin in 5% CO2 at 37°C. Additional details are provided in ***Supplementary Detailed methods.***

### Isolation of LSCs and gene expression analysis

The cells were analyzed and sorted using the MoFlo FACS system (XDP, Beckman), and LSCs were identified inside the area of CD34+ and CD38-. FACS and trypan blue staining detected the enrichment of LSCs. Human Signal Transduction Pathway FinderTM RT2 ProfilerTM PCR Array (PAHS-014A, Qiagen, USA) was used to screen the differences in 84 key genes that are representative of 18 different signal transduction pathways between LSCK562 and LSCK562/ADM. Additional details are provided in ***Supplementary Detailed methods.***

### Cell viability, apoptosis assays, and FACS analysis

Cell viability was assessed indirectly using MTT assay. Apoptosis assay was detected by Annexin V/PI double staining. FACS was used to analyze cell cycle distribution, ROS generation, P-gp drug pump activity, activated caspase 3 and P-gp relative expression. A detailed description of the experimental procedures was available in the ***Supplementary Detailed methods.***

### Wright-Giemsa staining and electron microscope (EM)

All the treated cells were stained with Wright-Giemsa and observed under AX80 optical microscope (Olympus, Tokyo, Japan). The cellular ultrastructure was observed under a JEM1230 transmission electron microscope (JEOL, Japan). Additional details are provided in ***Supplementary Detailed methods.***

### Methylcellulose colony formation assay

A total of 1000 cells were plated in methylcellulose culture media supplemented with 0.9% methylcellulose, 20% fetal bovine serum, 1mM l-glutamine, 50Uml-1 penicillin, and 50Uml-1 streptomycin and 5μM 2-mercaptoethanol in the presence of different concentrations of PTL. Clones containing more than 40 cells were scored after culturing for 10 days at 37°C in 5% CO2.

### Quantitative real-time PCR (RT-PCR)

The mRNA levels of *mdr-1, bcrp* and *bcl-2* were evaluated using RT-PCR by a Rotor-Gene 3000 quantitative PCR amplifier (CobetteRes. Inc, Sydney, Australia). Additional details are provided in ***Supplementary Detailed methods.***

### Nuclear extraction

Cell nuclear fractions were prepared from K562/ADM cells according to the *Nuclear and Cytoplasmic Protein Extraction Kit* manufacturer. The cells were resuspended in 200 μl Cytoplasm Extraction Buffer A, vortexed intensely for 5 s, and then incubated at 4°C for 15 min. The cytoplasmic proteins extraction buffer B was added, vortexed for 5 s and then incubated for 1 min on ice. After centrifuging for 5 min at 14,000 g at 4°C, the supernatant was removed, and the pellet was resuspended in the nuclear protein extraction buffer. After incubating for 30 min on ice and centrifuging for 10 min at 4°C, the supernatants containing the nuclear fraction were obtained.

### Statistical analysis

All numerical data were presented as means ± S.D from at least three independent experiments. The differences between control and experimental groups were analyzed using Student's *t*-test. *P* <0.05 was considered to be statistically significant.

## Results

### Identification of drug-resistant leukemic stem cells

**Figure 1:**
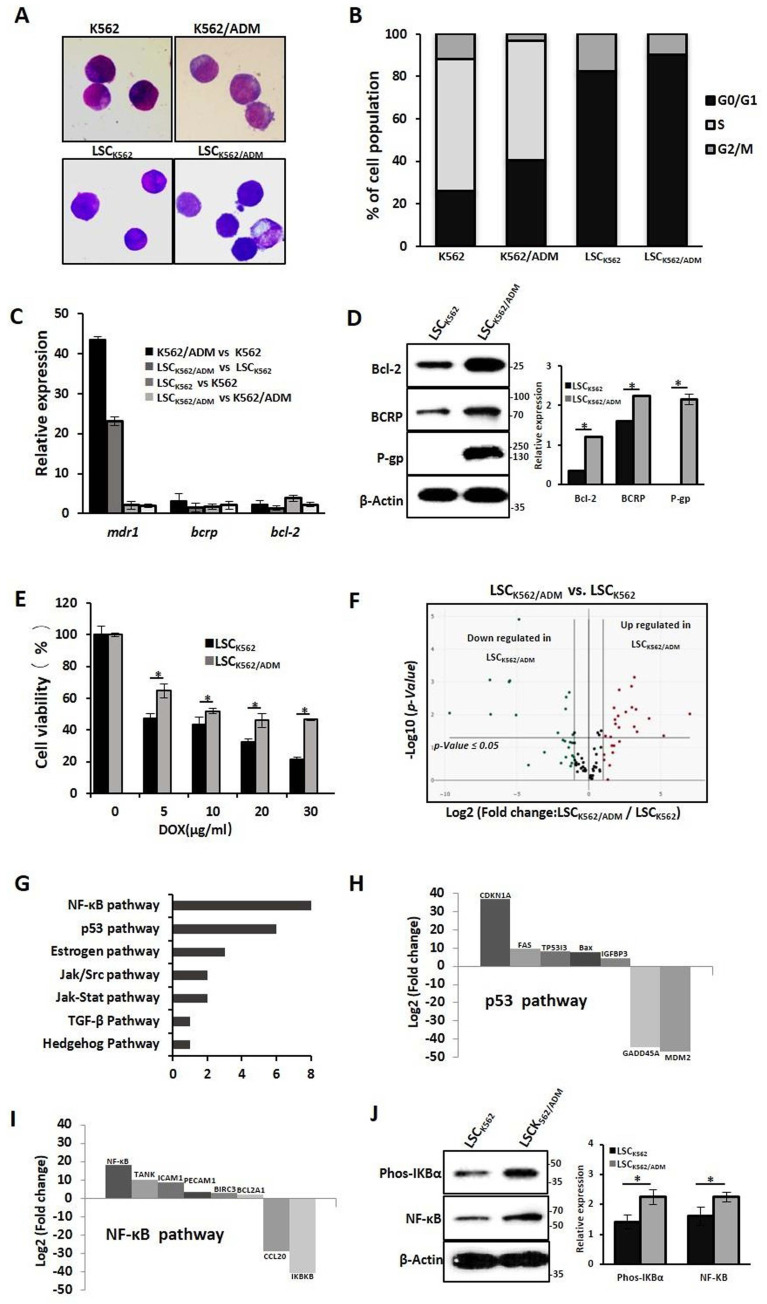
** Characterizations of drug-resistant leukemia stem cells (LSC_K562/ADM_). (A)** The morphology of cells was observed using a phase-contrast microscope after Wright-Giemsa staining(Magnification × 20). **(B)** The distribution of cell cycle was analyzed using FACS. **(C)** RT-PCR analysis of *mdr-1*, *bcrp* and *bcl-2*. **(D)** Western blot analysis of P-gp, BCRP and Bcl-2. Right panel shows quantification of indicated proteins level (*P < 0.01, versus LSC_K562_ group). **(E)** LSC_K562_ and LSC_K562/ADM_ were treated with indicated concentration of DOX for 24h, MTT assay assessed the survival rate of cells (*P < 0.01, versus LSC_K562_ group). **(F)** Volcano plot of genes expressed differentially between LSC_K562_ and LSC_K562/ADM_. Vertical lines indicate -Log10 fold change. A horizontal line indicates *P* = 0.05 threshold. **(G)** shows that the number of differential expression genes in 7 signal transductions. **(H)** and **(I)** Genes that changed at least a two-fold differential expression in p53 pathway and NF-κB pathway. **(J)** LSC_K562_ and LSC_K562/ADM_ were collected and subjected to western blot with indicated antibodies. Right panel shows quantification of indicated proteins level (*P < 0.01, versus LSC_K562_ group).

Our previous studies have confirmed that LSCs with high expression of P-gp and BCRP might be the causes of drug resistance in K562/ADM cells^13^. To further study the biological characteristics of drug-resistant LSCs, LSCs from K562 cells and K562/ADM cells were isolated by flow cytometry and immunological marker CD34+CD38- on LSCs, and named these cells as LSCK562 and LSCK562/ADM, respectively. Next, whether these isolated cells have stem-cell-like properties was assessed. The results of Wright-Giemsa staining revealed the ratio of nucleus to cytoplasm cut down in LSCK562 and LSCK562/ADM (Fig. 1A). Cell cycle analysis indicated about 90% proportion of G0/G1 phase, and disappearance of S phase in LSCK562 and LSCK562/ADM (Fig. 1B). RT-PCR showed that the mRNA levels of anti-apoptotic gene (*bcl-2*) and ABC transporters (*bcrp* and *mdr1*) in isolated LSCs were much higher than that in total population (Fig. 1C). These results suggested that isolated cells possess the characteristics of stem cells, and these cells can be subsequently used to explore the features of drug-resistant LSCs.

Next, the differences between LSCK562 and LSCK562/ADM were investigated. At first, compared with LSCK562, mRNA levels of *bcl-2*, *bcrp* and *mdr1* demonstrated a significant enhancement in LSCK562/ADM (Fig. 1C), and western blotting analysis also showed similar results (Fig. 1D). MTT was used to detect the sensitivity of LSCs to a traditional drug (doxorubicin, DOX), and the results revealed that LSCK562/ADM was more tolerant than LSCK562 (Fig. 1E). After that, RT^2^ Profiler PCR Array Human Signal Transduction Pathway Finder chip was used to screen the changes of 18 signal transduction pathways including 98 genes that were involved in survival and death in LSCK562 and LSCK562/ADM and identified the presence of 30 genes in 7 signal pathways changed in LSCK562/ADM (Fig. 1F and 1G). Further analysis of these results showed that p53-dependent cell cycle arrest was more significant in LSCK562/ADM (Fig. 1H). Moreover, cell cycle analysis showed that the proportion of G0/G1 was up to 90.41% in LSCK562/ADM, which was higher than that in LSCK562 (Fig. 1B). Also the NF-κB pathway was aberrantly activated in LSCK562/ADM (Fig. 1 I), and western blotting analysis also exhibited similar consequences (Fig. 1J). So, we decided to hunt for some active compounds to eradicate LSCK562/ADM to provide differences between LSCK562 and LSCK562/ADM, yielding a strategy for the treatment of chemo-resistance and relapse of leukemia.

### PTL induced K562/ADM cells apoptosis by increasing ROS, attenuating NF-κB activation and activating mitochondrial-dependent pathway

**Figure 2:**
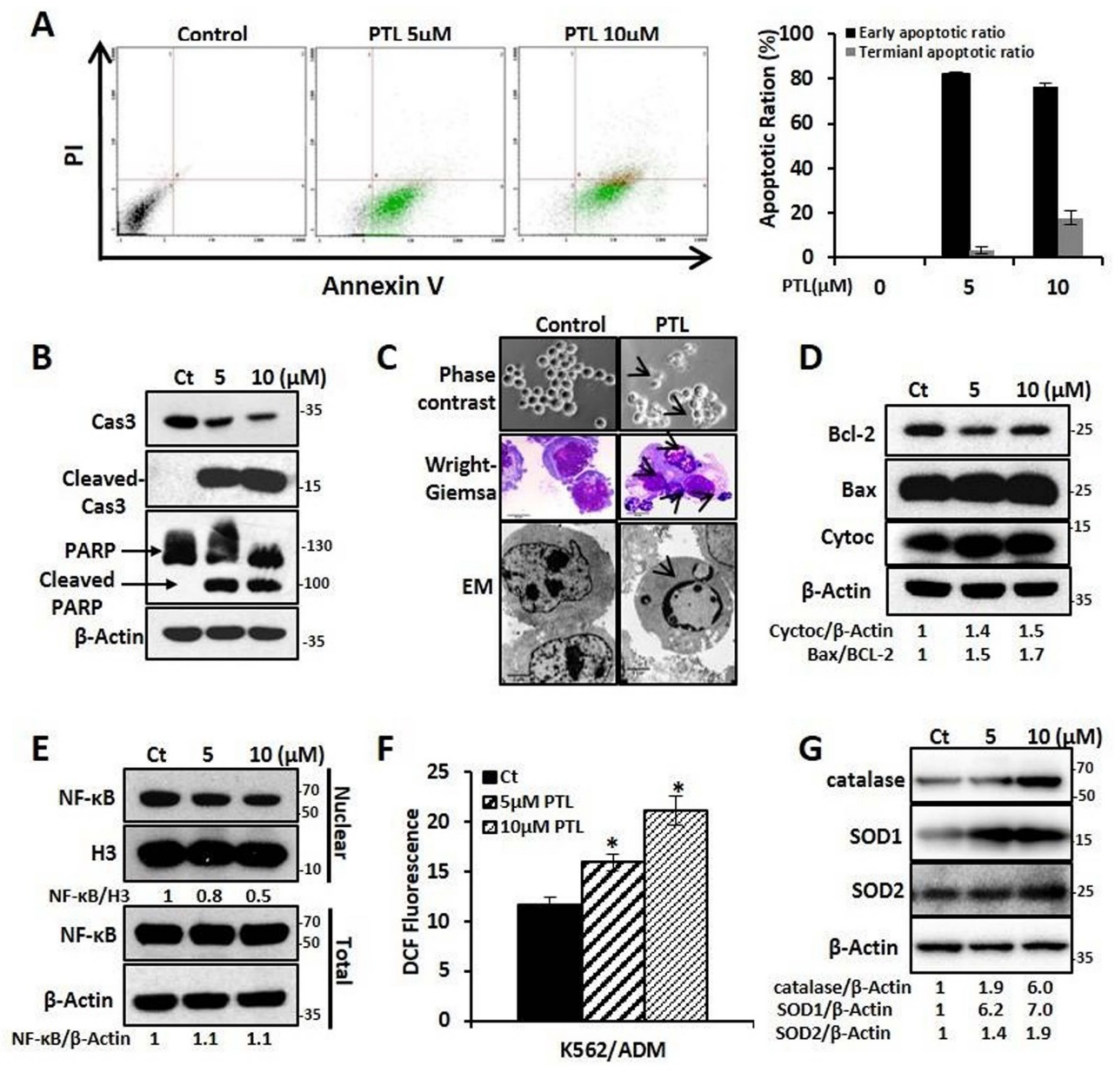
** PTL induced K562/ADM cells apoptosis dependent on mitochondria pathway and NF-κB pathway.** K562/ADM cells were treated with 5μM and 10μM PTL for 24h. **(A)** Cells labeled with Annexin V/PI were analyzed by FACS. Left panel shows a typical flow cytometry plot. Right panel represents histograms of all data. **(B)** Cells were analyzed by western blot with indicated antibodies. **(C)** The changes of morphology were observed by phase-contrast microscope (Magnification × 20), electron microscope (EM, Magnification × 10,000 scale bar, 2μm) and Wright-Giemsa staining (Magnification × 100, scale bar, 20μm). The arrowhead (↓) indicates the chromatin condensation, nuclear fragmentation and apoptotic bodies in apoptotic cells. **(D)** Cells were subjected to western blot with indicated antibodies. **(E)** Nuclear fractions were fractionated from the treated cells,proteins were detected by western blot with the indicated antibodies. **(F and G)** Cells were subjected to FACS analysis using DCFH-DA probe (*P < 0.01, versus control group) or western blot with indicated antibodies.

In this study, the effect of PTL on K562 and K562/ADM, LSCK562, and LSCK562/ADM was evaluated. Firstly, MTT assay showed that PTL inhibited cell proliferation in a dose- and time-dependent manner after treatment with PTL. The IC50 values at 24 h and 48 h demonstrated no noticeable differences between K562 and K562/ADM, LSCK562 and LSCK562/ADM (18.57μMand 7.52μM in K562, 19.75μM and 8.55μM in K562/ADM; 32.45μM and 21.33μM in LSCK562, 32.37μM and 26.01μM in LSCK562/ADM, respectively) (Supplemental Fig. S1A and Fig. S1B). These results indicated that PTL, unlike other traditional chemotherapeutic drugs, suppressed cell growth without any difference in sensitive cells and drug-resistant cells. Next, the results of apoptotic analysis revealed that treatment with PTL significantly increased the apoptotic rates of K562/ADM cells, and predominantly the early apoptosis (Fig. 2A). Western blotting analysis further verified this result by detecting the cleavage of caspase 3 and PARP. Finally, the morphological changes of K562/ADM cells were detected using Wright-Giemsa staining, phase contrast microscopy, and electron microscopy. These results showed visible apoptotic morphological changes after PTL treatment, such as nuclear pyknosis and the appearance of apoptotic bodies (Fig. 2C). These results suggested that PTL could efficiently induce apoptosis in K562/ADM cells.

To further explore the mechanism of PTL-induced apoptosis in K562/ADM cells, the related molecules of the apoptotic pathway were further examined. After treating the cells with 5μM and 10μM PTL for 24h, western blotting analysis showed that the intracellular Bax/Bcl-2 ratio was increased to 1.5 and 1.7 times of the control group; the expression of cytochrome C was increased to 1.4 and 1.5 times that of the control group (Fig. 2D); and the levels of NF-κB in the nucleus were decreased by 0.8 times and 0.6 times that of the control group, with no significant changes in the whole-cell (Fig. 2E). Moreover, the ROS levels were increased to 37% and 81.9% in the control group (Fig. 2F) and the expression levels of the three antioxidant enzymes (catalase, SOD1 and SOD2) were significantly enhanced (Fig. 2G). These results confirmed that PTL could induce apoptosis of K562/ADM cells through mitochondrial-dependent signaling pathway by increasing the ROS levels and inhibiting the activity of NF-κB.

### PTL potentiated DOX-induced apoptosis of K562/ADM cells by NF-κB pathway-mediated down-regulation of P-gp expression and activity

**Figure 3:**
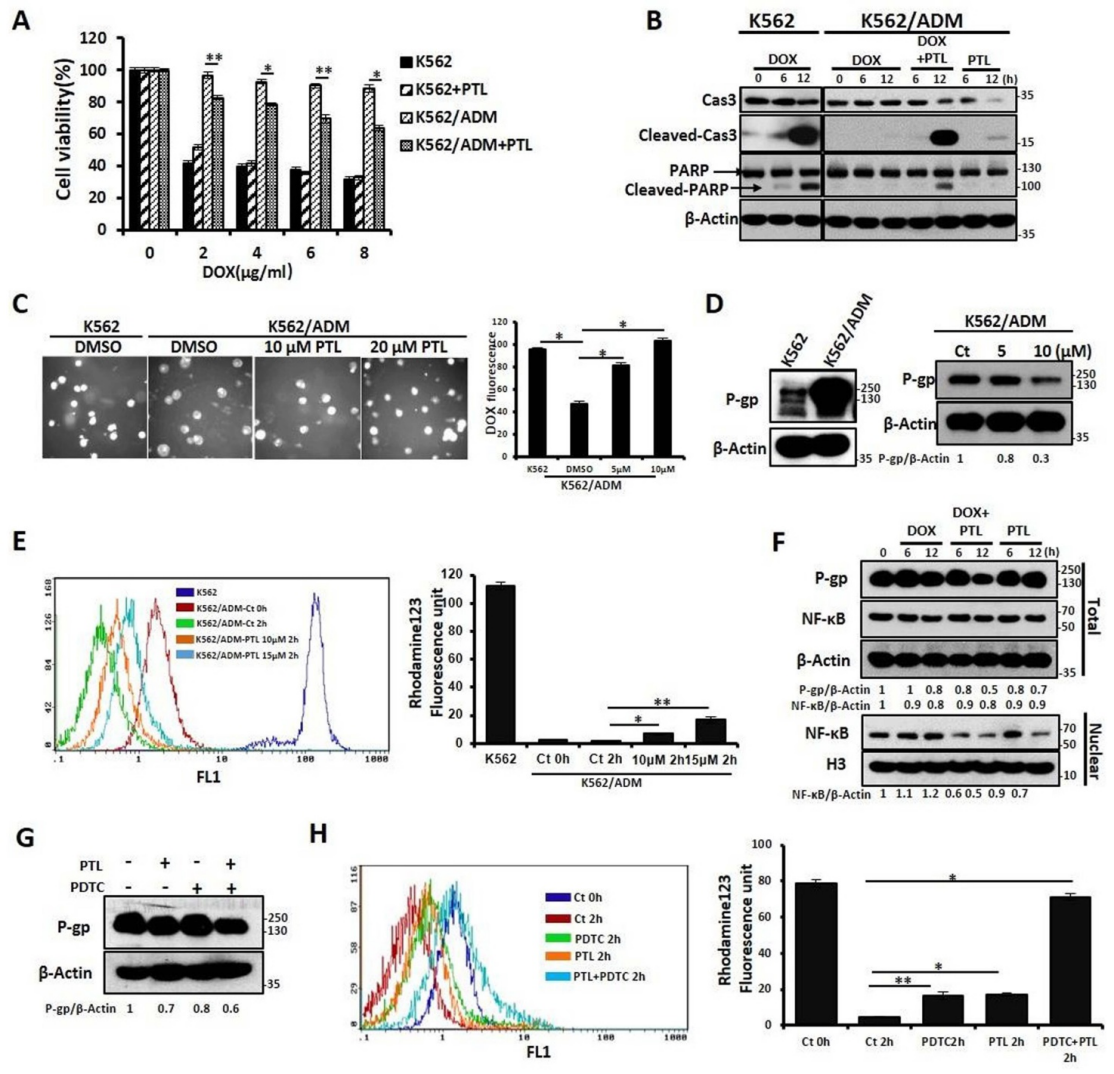
** PTL enhanced the sensitivity to DOX through down-regulating expression of P-gp caused by inhibition of NF-κB pathway. (A)** Different doses of DOX treated K562 cells, and K562/ADM cells with or without 10μM PTL for 24h, the survival rate of cells was assessed by MTT assay (*P < 0.01, **P < 0.05, versus untreated K562/ADM group). **(B)** K562 cells and K562/ADM cells were treated with DOX, PTL or DOX plus PTL for 6h and 12h respectively, indicated proteins were detected by western blot. **(C)** 2μM DOX treated K562 cells and K562/ADM cells with or without indicated concentrations of PTL for 6h, the content of DOX were analyzed by a fluorescence microscope (Magnification × 20). Right panel shows quantification of DOX fluorescence (*P < 0.01). **(D)** K562 cells and K562/ADM cells were collected and subjected to western blot analysis (left panel). K562/ADM cells were treated with 5μM and 10μM PTL for 12h, indicated proteins were detected by western blot (right panel). **(E)** K562 cells and K562/ADM cells were treated with or without 10μM and 15μM PTL for 12h, and then were collected and treated as described in ***Materials and Methods***. Left panel shows a typical flow cytometry plot. Right panel represents histograms of all data (*P < 0.01, **P < 0.05). **(F)** Nuclear fractions were extracted from K562/ADM cells that were treated with DOX, PTL or DOX plus PTL for 6h and 12h respectively, and then western blot was performed to detect the indicated proteins. **(G)** K562/ADM cells were treated with 10μM PTL for 6h. Cells were treated with PTL and/or 2μM PDTC (an inhibitor of NF-κB) for 2h before harvesting and were subjected to western blot using indicated antibodies. **(H)** The activity of P-gp was assessed after treated cells as in (G) (*P < 0.01, **P < 0.05).

Our previous results confirmed that K562/ADM cells not only have a high resistance to DOX but also have cross-tolerance to other structural types of conventional antineoplastic drugs (DNR, Vp16). However, in this study, PTL significantly reduced the tolerance of K562/ADM cells to DOX, without influencing K562 cells (Fig. 3A and 3B, Supplemental Fig. S2A and 2B). Next, the mechanisms of PTL sensitization of K562/ADM cells to DOX were explored. For the antitumor effects of drugs, the accumulation of drugs in a cell remains fundamental. So, fluorescence microscopy showed that intracellular DOX content was increased gradually with increased PTL doses (Fig. 3C).

P-gp protein sharply declines the cellular accumulation of chemotherapeutic drugs, and high expression of P-gp protein is one of the main factors of MDR in K562/ADM cells. Western blotting results showed that the expression of P-gp on K562/ADM cells remained high when compared with sensitive K562 cells. However, PTL treatment of K562/ADM cells revealed dramatic decrease of the protein levels of P-gp through western blotting and flow cytometry analyses (Fig. 3D, Supplemental Fig. S2C). In the following experiments, the activity of P-gp utilizing the spontaneous green fluorescence Rhodamine123 that was used to simulate the intracellular entry and exit of chemotherapeutic drugs was assessed. The results showed that PTL increased Rhodamine 123 residue in cells, suggesting that the P-gp activity was inhibited by PTL (Fig. 3E). Finally, to investigate whether PTL-mediated down-regulation of P-gp depends on NF-κB signaling pathway, western blotting was performed to analyze the correlation between P-gp and nuclear NF-κB. The data showed that the decrease in P-gp was consistent with the down-regulation of NF-κB expression in the nucleus, especially in the DOX plus PTL group. However, the expression of NF-κB was slightly enhanced in DOX group (Fig. 3F). Pyrrolidine dithiocarbamate (PDTC), a reported inhibitor of NF-κB, was employed to further confirm that NF-κB pathway was responsible for the recession of P-gp. Western blotting, FACS and RT-PCR analyses showed that PDTC combined with PTL accelerated the declination of P-gp (Fig. 3G, Supplemental Fig. S2D and Fig. S2E), leading to the accumulation of DOX in K562/ADM cells (Fig. S2F). Rhodamine 123 evaluated the function of P-gp, and the results showed that the combined action of PDTC and PTL could significantly increase the residue of Rhodamine 123 in cells (Fig. 3H).

These results confirmed that PTL reduced the expression of P-gp in K562/ADM cells by inhibiting the activity of NF-κB and weakened the function of P-gp, leading to increased concentrations of chemotherapeutic drugs to promote apoptosis in drug-resistant cells.

### PTL increased apoptosis and down-regulated P-gp expression in LSCK562/ADM cells by attenuating abnormal NF-κB activity

**Figure 4:**
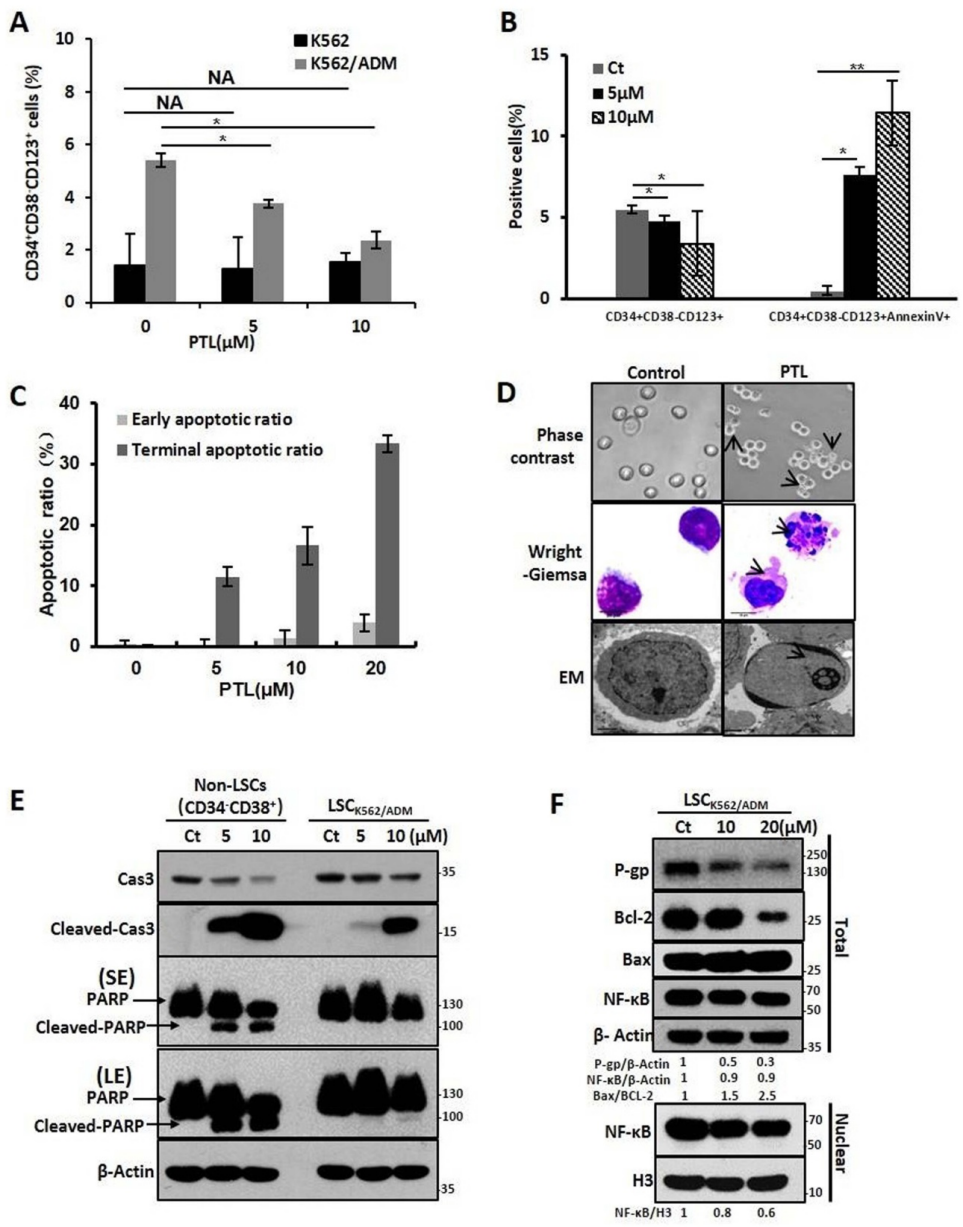
** PTL significantly eradicated drug-resistant LSCs by inducing cells apoptosis. (A)** K562 cells and K562/ADM cells were treated with 5μM and 10μM PTL for 24h and harvested for FACS using a marker in LSCs (CD34^+^CD38^-^CD123^+^) (*P < 0.01, versus control group). **(B)** K562/ADM cells were treated as in (A) and analyzed using FACS combined with Annexin V and marker in Leukemia stem cells(CD34^+^CD38^-^CD123^+^). **(C)** LSC_K562/ADM_ were treated with indicated concentrations of PTL for 24h. Cells were labeled with Annexin V/PI and assessed the apoptotic rate using FACS (*P < 0.01, **P < 0.05, versus control group). **(D)** LSC_K562/ADM_ were collected after treatment with 20μM PTL for 24h, and then observed the changes of morphology by phase-contrast microscope (Magnification × 20), electron microscope (EM, Magnification × 10,000, scale bar,2μm) and wright-Giemsa staining (Magnification × 100, scale bar, 20μm). The arrowhead (↓) indicates the chromatin condensation, nuclear fragmentation and apoptotic bodies in apoptotic cells. **(E)** LSCK562/ADM and Non-LSCs (CD34-CD38+) were treated with 5μM and 10μM PTL for 24h and harvested for western blot using indicated antibodies. **(F)** LSC_K562/ADM_ were treated with 10μM and 20μM PTL for 12h, the nuclear fractions were fractioned and subjected to western blot with indicated antibodies.

Next, we investigated whether PTL inhibited and killed LSCK562/ADM cells. So, the relative proportion of CD34+CD38- CD123+ that was reported as the surface marker of LSCs in K562/ADM cells was assessed. The data elucidated that PTL could remarkably decline the relative amount of CD34+CD38- CD123+ in K562/ADM cells after PTL treatment, but no significant changes were observed in K562 cells because of less proportion of LSCs in K562 cells (Fig. 4A). Next, combining with unique surface markers, the apoptotic rate of LSCs (CD34+CD38- CD123+Annexin V+) in K562/ADM cells was also increased significantly (Fig. 4B), suggesting that PTL could induce apoptosis of LSCs in K562/ADM. To further confirm the apoptotic induction of PTL on LSCs derived from K562/ADM, LSCs were isolated from K562/ADM cells, and i.e., from LSCK562/ADM. After PTL treatment, Annexin V/PI staining revealed that the apoptotic rate of LSCK562/ADM was gradually enhanced after treatment with different concentrations of PTL (Fig. 4C). The morphological changes of LSCK562/ADM caused by PTL were observed by Wright-Giemsa staining, phase contrast microscopy and electron microscopy. These results showed noticeable morphological changes of apoptotic cells after PTL treatment, such as nuclear pyknosis and the appearance of apoptotic body (Fig. 4D). Next, western blotting analysis was revealed that PTL decreased the levels of caspase 3 and increased the levels of activated caspase 3 and PARP in differentiated leukemia cells (CD34^-^CD38^+^) and LSCK562/ADM (CD34^+^CD38^-^). Nevertheless, compared with differentiated cells, LSCK562/ADM cells were less sensitive to PTL (Fig. 4E). At the same time, the effects of PTL on apoptotic pathway proteins of LSCK562/ADM were examined. Western blotting results showed that the ratio of Bax/Bcl-2 in the cells was increased (Fig. 4F). The level of P-gp in LSCK562/ADM was also higher than that in LSCK562 and matured K562/ADM cells, and PTL treatment could weaken the expression of P-gp (Fig. 4F). Similarly, after LSCK562/ADM were treated with PTL, the content of NF-κB in the nucleus was decreased by 0.8 and 0.6 times that of the control group, which was consistent with the down-regulation of P-gp in the total cells (Fig. 4F). These results suggested that PTL treatment weakens the P-gp protein levels by inhibiting the activity of NF-κB and inducing apoptosis of LSCK562/ADM through mitochondrial-dependent signaling pathway.

### PTL suppressed self-renewal of LSCs

**Figure 5:**
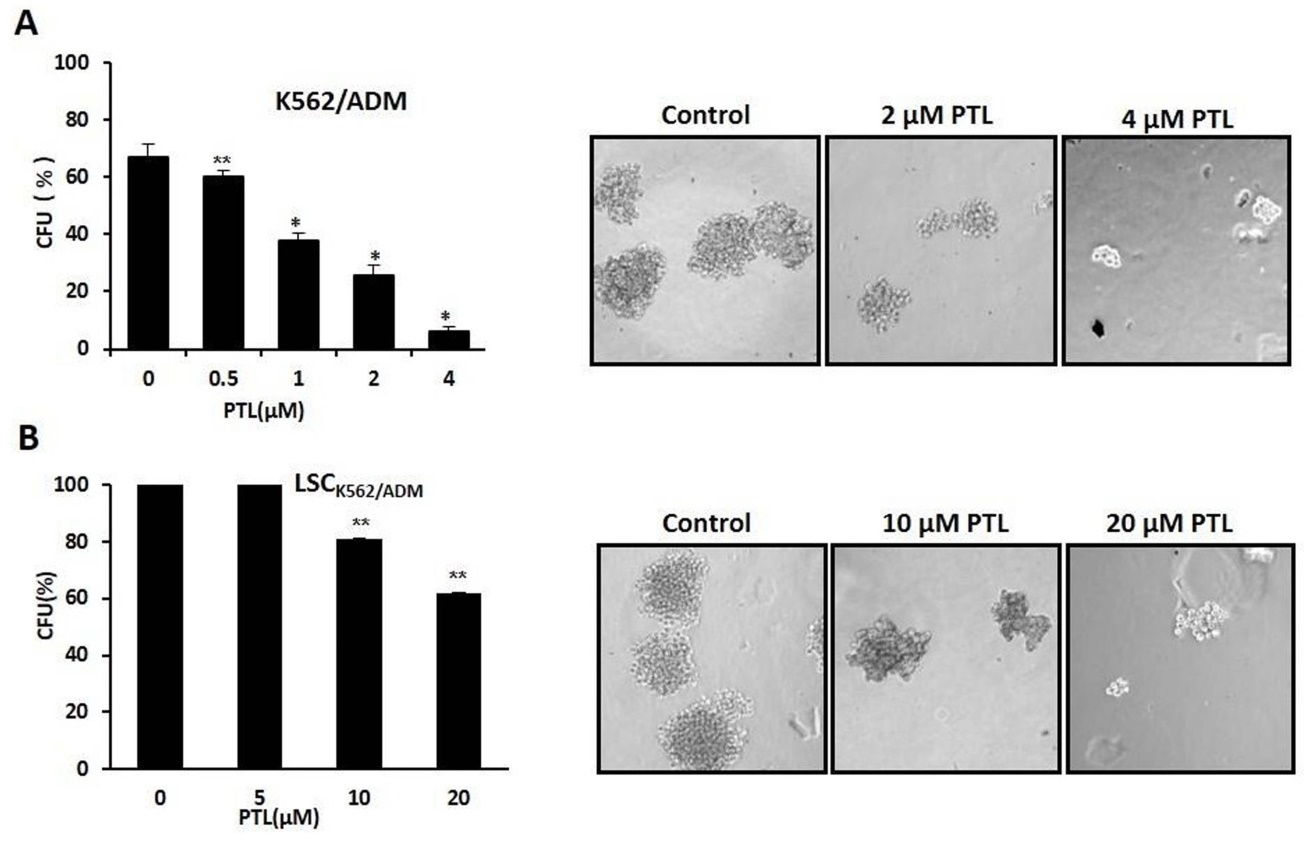
In vitro clone assay for K_562/ADM_ cells and LSC_K562/ADM_ cells after treatment with PTL. **(A)** 0.5μM ~4μM PTL was directly added to the methylcellulose culture in presence of K562/ADM cells, and cells were cultured for 10 days. The clones were observed and counted under a light microscope. **(B)** LSC_K562/ADM_ cells directly treated with 5μM~20μM PTL in methylcellulose culture for 10 days, and then clones were observed and counted under a light microscope. Significant difference was compared to control (*P <0.01, **P <0.05). Left panel represents histograms of all data. Right panel shows the shapes of colons after different treatments (Magnification × 10).

Colony formation assay was performed to assess the self-renewability of LSCs as described previously by Smyth MJ et al. 30. Briefly, 200 cells/ml were cultured in methylcellulose hemi-solid culture in the absence or presence of PTL. Colonies with over 40 cells as colony formation units (CFUs) were scored and observed after 10 days. Firstly, treated K562/ADM cells with 0-4μM PTL showed reduction of CFUs by 90.56% at 4 μM when compared with control (Fig. 5A). Next, isolated LSCK562/ADM cells were treated with 0-20μM PTL, and CFUs were reduced by 40% (Fig. 5B). These results suggested that PTL treatment had a striking effect on K562/ADM cells with self-renewability and high proliferation potential, such as LSCs.

## Discussion

LSCs that are naturally resistant to clinical chemotherapeutic drugs play a vital role in MDR. However, it is still unknown regarding the features of LSCs in drug-resistant leukemia and relapsed leukemia. According to our findings, the concept of “drug-resistant LSCs” exhibited that drug-resistant LSCs existed in drug-resistant leukemia and relapsed leukemia, and also a more significant population with higher anti-apoptotic proteins and ABC transporter proteins than LSCs in sensitive leukemia and untreated leukemia^13^. Therefore, it is considered as an important topic to explore and identify the specific features of drug-resistant LSCs. In our study, drug-resistant LSCs and sensitive LSCs were isolated from K562/ADM cells and K562 cells, respectively. Next, using gene chip analysis, NF-κB signaling pathway that was abnormally activated in drug-resistant LSCs was identified. NF-κB is a vital complex that controls the transcription of DNA and cell survival, and incorrect regulation of NF-κB is associated with cancer and viral infection. So, exploration of natural compounds that inhibit the activity of NF-κB contributes to the clearance of drug-resistant LSCs. However, many traditional chemotherapeutics have promoted nuclear transfer of NF-κB and activated its downstream anti-apoptotic and MDR genes while exerting anti-cancer function^31^, ^32^. PTL is the active ingredient of feverfew. In addition to its strong anti-infective ability, extensive studies have confirmed that PTL is an effective anti-cancer drug and has no severe side-effects^33^. At present, a phase I trial of PTL on patients with all kinds of cancers has been conducted^34^, ^35^. Also, PTL exhibited distinct inhibition of LSCs^36^, and therefore the clinical application of PTL should be safer and promising in patients with cancers.

In the following experiments, the number of LSCs in K562/ADM cells was 4.12 times higher than that of LSCs in K562 cells, but PTL could efficiently clear away these two LSCs by inducing apoptosis with no differences at all. As for the mechanism of induction of K562/ADM cells and drug-resistant LSCs apoptosis, our results showed that PTL inhibited the transcriptional activity of NF-κB and enhanced generation of reactive oxygen species (ROS). Besides, PTL also significantly dampened the self-renewability of LSCK562/AMD cells, which might be caused due to the inhibition of abnormal activation of NF-κB signaling pathway. These results suggested that PTL might act as a hopeful agent for eliminating drug-resistant LSCs.

P-gp is a kind of transmembrane protein that belongs to ABC transporter family, and it can pump the chemotherapeutic drugs out of cancer cells. In this study, both K562/ADM resistant cells and drug-resistant LSCs showed high expression of P-gp. Furthermore, PTL treatment weakens the expression and activity of P-gp, promotes the accumulation of DOX in cells and decreases the tolerance of cells to drugs. In the follow-up experiments, the reason for the effect of PTL expression and activity of P-gp was investigated. Our data showed that down-regulation of P-gp was consistent with the reduction of nuclear transfer of NF-κB. Therefore, we speculated that NF-κB , as a transcription factor, might regulate P-gp expression in K562/ADM cells and drug-resistant LSCs. Furthermore, another effective inhibitor of NF-κB, PDTC, was used to confirm this result. The data demonstrated that PDTC down-regulated the expression of P-gp, and PTL combined with PDTC has more apparent effects. Besides, Ge Zhou et al. 31 have demonstrated that the transcription factor of NF-κB mediates insulin-induced *mdr1b* expression. So, PTL treatment suppressed the transcriptional activity of NF-κB, declining the expression of P-gp. However, the mechanism of PTL affecting NF-κB pathway in the regulation of expression of P-gp was not explained. In future studies, this should be investigated and discussed. Our data also found that the effect of PTL on LSCs was significantly lower than that on differentiated and mature leukemia cells, which might be associated with different features of the two types of cells, and much in depth analysis has been done in this study.

All in all, our results confirmed that PTL induced apoptosis of drug-resistant K562/ADM cells and their LSCs, namely the drug-resistant LSCs, through mitochondrial-dependent signaling pathway, and potentiated the sensitivity of K562/ADM cells to doxorubicin-induced apoptosis via down-regulation of P-gp function mediated by NF-κB signaling pathway. The precise effects and mechanisms of PTL requires in-depth studies *in vivo* xenograft model and leukemia patient-derived xenograft (PDX) model, and such a procedure will be performed in the on-going experiments to thoroughly explore and comprehensively hunt for the potential of PTL's in anti-drug-resistant leukemia. This study likely provides valid therapeutic targets for the treatment of clinical refractory and relapsed leukemia.

## Supplementary Material

Supplementary methods and figures.
