# Supplementary material for: Suppression Of Aberrant Activation Of NF-κB Pathway In Drug-resistant Leukemia Stem Cells Contributes To Parthenolide-potentiated Reversal Of Drug Resistance In Leukemia
Source: J Cancer. 2021 Jul 25;12(18):5519–29. doi: 10.7150/jca.52641 (PMC8364658; doi:10.7150/jca.52641)
Supplement: Supplementary file 1 — Supplementary methods and figures. [file jcav12p5519s1.pdf]

Supporting information

Detailed methods

Regents and antibodies

Regents: PTL (P8522f) was purchased from BIOMOL. Doxorubicin (D1515), PDTC (P8765) and DCFH-DA (D6883) were obtained from Sigma-Aldrich. Rhodamine123 (R8030), MTT (M8180) and PI (C0080) were obtained from Solaibio. Annexin V/PI staining kit was from eBioscience (800-8005-72). Methylcellulose 4000CPS）was from AMRESCO (K390). Nuclear Extraction kit was provided by Beyotime (P0027). Primary antibodies we used were Bcl-2 (Cell Signaling Technology, 15071), Bax(Cell Signaling Technology, 2772), BCRP (Cell Signaling Technology, 42078), P-gp (Cell Signaling Technology, 13978), Phoso-IKBα (Santa Cruze, 8404), β-Actin (Immmunoway, YM3028), Caspase3 (Cell Signaling Technology, 9662), Cleaved Caspase3 (Cell Signaling Technology, 9661), PARP (Cell Signaling Technology, 9532), Cleaved PARP (Cell Signaling Technology, 5625), Cytochrome c (Cell Signaling Technology, 11940), SOD1 (Santa Cruze, SC11407), NF-κB (Santa Cruze, SC8008), Histon H3 (Cell Signaling Technology, 4499), catalase (Santa Cruze, SC50508), SOD1 (Santa Cruze, SC11407), SOD2 (Santa Cruze, SC30080). Secondary antibodies were all from Jackson Immunoresearch Laboratories and included anti-mouse- HRP (115-005-003) and anti-rabbit-HRP (111-035-003).

Cell culture

Doxorubicin-induced and P-glycoprotein-overexpressed multidrug-resistant human leukemia cells (K562/ADM cells) and sensitive cells (K562 cells) were cultured in RPMI1640 (Gibco, Thermo Fisher Scientific, USA) medium supplemented with 10% fetal bovine serum (FBS, GE Healthcare Life Sciences, USA), 1mM l-glutamine, 50Uml^{-1} penicillin, and 50Uml^{-1} streptomycin. The cell cultures were maintained in an incubator at 37°C and 5% CO_2. To maintain drug resistance, doxorubicin (4.0 mgL^{-1}) was supplemented at regular intervals. Cells could be harvested and used for experiments 1

week after the removal of doxorubicin.

Isolation of the LSCs from K562/ADM and K562 cell line by FACS

1×10^8 cells were collected and washed with cold PBS three times. To isolate LSCs from K562/ADM cells and K562 cells, a single-cell suspension of 1×10^6 ml^{-1} was prepared and stained with anti–CD34-PE-CY5 and Anti–CD38-ECD (Caltag Lab, USA for 15 min in the dark at room temperature. Next, the cells were washed 3 times with cold PBS and were suspended in cold PBS with 2% FBS. The following filtration using 400 mesh strainer, the cells were analyzed and sorted by FCM using the MoFlo system (XDP, Beckman). The cells inside the area of $CD34^+$ and $CD38^-$ were identified as leukemia stem cells (LSCs). The enrichment of LSCs was detected by FACS and trypan blue staining.

Gene expression analysis

Human Signal Transduction Pathway Finder^{TM} RT^2 Profiler ^{TM} PCR Array (PAHS-014A, Qiagen, USA) was used to screen the differences of 84 key genes representative in 18 different signal transduction pathways between LSC_{K562} and $LSC_{K562/ADM}$. Five housekeeping genes (Actb,B2m,Hrpt1,Ldha and Rp1p1) were used for normalization of samples. The data and volcano plot were analyzed and produced using the RT^2 array data analysis web portal (https://dataanalysis2.qiagen.com/pcr).

Proliferation assay

The cells were cultured in the 96-wells plates at a density of $1\times10^5 ml^{-1}$and treated with different concentrations of PTL for 24 h-48 h. MTT was added to each well before termination of culture and incubated for 4 h at 37°C in 5% CO_2. Then, 10%SDS was be added to each well, followed by overnight incubated at 37°C and 5% CO_2 to dissolve the dark blue crystal product. Each sample point was assayed with 4 replica points. Absorbance at 570 nm (A570) of the solubilized formazan was measured using a Bio-Tek Instruments (KC junior, USA) microplate reader to calculate inhibition rate for cell proliferation and 50% inhibitory concentration (IC_{50}).

Apoptosis assay

The cells were treated with different concentrations of PTL for 24 h. Then 1×10^6cells were collected and washed with cold PBS. Subsequently, 100µl Annexin V-binding buffer, FITC-conjugated Annexin V and PI were used to stain the cells in each sample for 15 min at room temperature. FACS (Beckman-Coulter Epics XL, USA) was employed to measure the apoptosis rate in the early and terminal phase with 3 replica points.

To assay the apoptosis of LSC in K562/ADM and K562 cell population, the cells were treated with 5µM and 10µM PTL for 24h and marked with PEcy5-CD34, ECD-CD38 and PE-CD123 antibodies at room temperature for 20 min, and then labeled with FITC-Annexin V in 100µl Annexin V-binding buffer for 15min. The percentage of $CD34^+CD38^-CD123^+$ cells was detected by FCM, and then gated with $CD34^+CD38^-CD123^+$ to analyze the apoptotic (Annexin V^+) cells in $CD34^+CD38^-CD123^+$ population. Each sample point was analyzed with 3 replica points.

Wright-Giemsa staining and electron microscope

Wright-Giemsa staining was performed as follows: after designed treatments, cells were reseeded to a coverglass slide and stained with Wright-Giemsa at room temperature for 30min. Changes of morphology were observed using AX80 optical microscope (Olympus, Tokyo, Japan) For electron microscope, cells were fixed, stained, dehydrated, embedded, cut into thin sections with an ultramicrotome and then cell sections were picked up on copper grids. After post-staining, these sections were analyzed with a JEM1230 transmission electron microscope (JEOL, Japan).

Assess the number of LSCs in whole cells population

After designed treatments, cells were labeled with anti–CD38-FITC, anti–CD34-PE-CY5 (Caltag Lab, USA) and anti–CD123-PE. (eBioscience, USA) and incubated at room temperature for 20 min followed by analysis using FACS. The mean percentage of $CD34^+CD38^-CD123^+$ was calculated from 3 independent experiments.

Cell cycle analysis

Cells were collected and washed with cold PBS 3 times. And then, 70% of cold ethanol

was added gently to fix cells overnight at -20℃. Before cell-cycle distribution being analyzed by FACS, cells were washed 3 times with cold PBS and stained with PI for 30min at room temperature.

Quantitative real-time PCR

Total RNA was extracted from cells using TRIzol (Invitrogen) and cDNA was synthesized using 1μg RNA with KIT (RR047A, Takara Bio, Otsu, Japan). The mRNA level was evaluated by a Rotor-Gene 3000 quantitative PCR amplifier (CobetteRes. Inc, Sydney, Australia). β-actin was used as an internal control of RNA integrity. Real-time PCR was performed in triplicate. The primers were as follows: human β-*actin* (5'-TGCTCCTCCTGAGCGCAAGTA-3' and 5'-CCACATCTGCTGGAAGGTGGA-3'), human *mdr-1* (5'-CCCATCATTGCA ATAGCA GG-3' and 5'-GTTCAA ACTTCTGCTCCTGA-3'), human *bcrp* (5’-GCTGCAAGGAAAGATCCAAGT-3’ and 5'-TAGTTGTTGCAAGCCGAAGAG-3'), human *bcl-2* (5'-GAGATGTCCAGCCAGCTGCAC-3' and 5' -ACAGGGCGATGTTGTCACCA- 3').

Western blot analysis

After designed treatments, cells were harvested and lysed in Laemmli SDS buffer (62.5 mM Tris-HCL pH 6.8, 25% glycerol, 2% SDS, phosphatase inhibitor and proteinase inhibitor cocktail). The whole lysate were collected and subjected to SDS/PAGE. The proteins were transferred to PVDF membrane. After blocking with 5% non-fat milk in TBST for 1hr, the membrane was probed with primary antibodies and secondary antibodies and visualized using ECL with film or CLINX Hemiscope (QinXiang, China).

Assessment of ROS generation

Harvested cells were washed with cold PBS 3 times and incubated with 5μM DCFH-DA for 30 min at 37°C. The green fluorescence was detected by FACS. The mean fluorescence intensity was calculated from 3 independent experiments.

The activity of P-gp drug pump

Cells were harvested and washed with cold PBS 3 times and suspended in 37°C PBS with 2% FBS. After being incubated for 2h at 37°C in 5% CO_2 in presence of 1μM Rhodamine123, cells were washed with cold PBS 3 times, suspended in 37°C PBS with 2% FBS and incubated for 2h at 37°C in 5% CO_2 again in absence of Rhodamine123. Next, cells were washed with cold PBS 3 times and prepared as a single-cell suspension of 1×10^6 ml^{-1}. Finally, the mean fluorescence intensity of Rhodamine123 in cells were analyzed by FACS in 3 independent experiments.

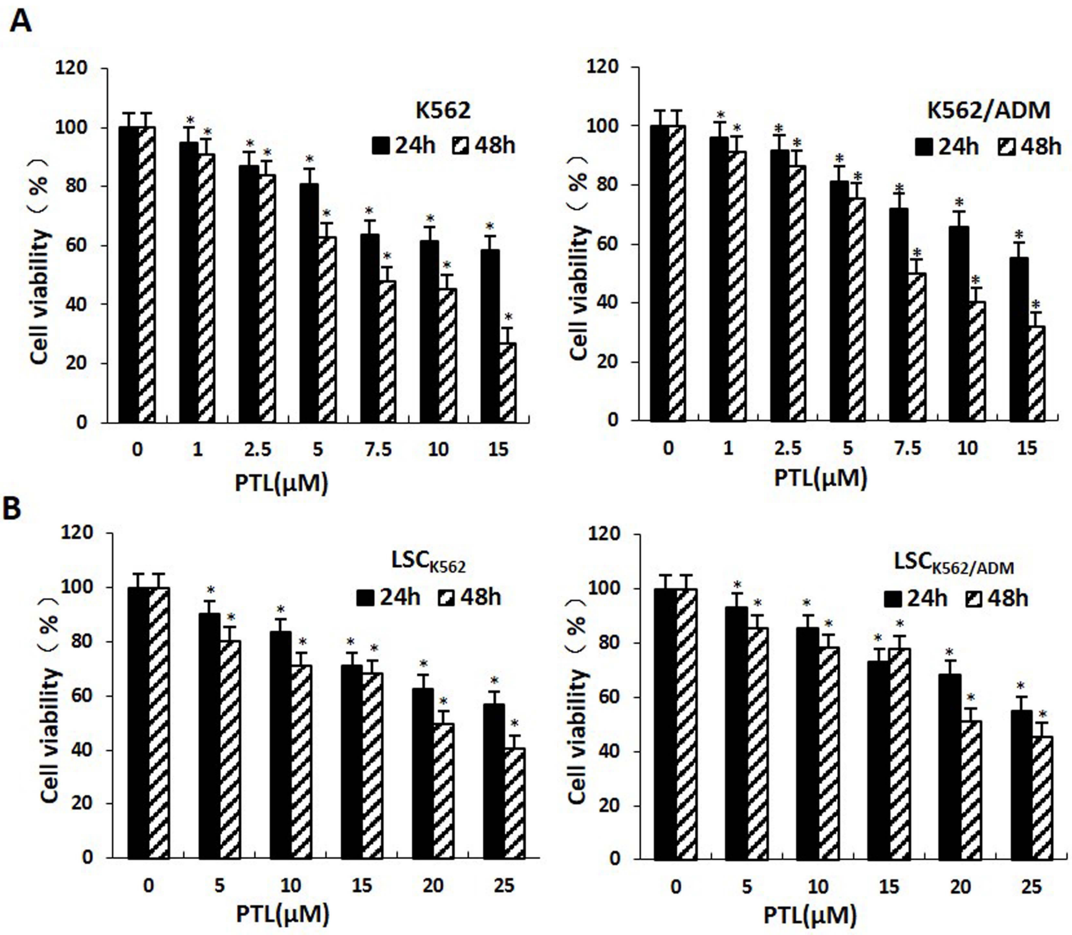

FigureS1.PTL suppressed the proliferation of K562 and LSC_{K562} cells, K562/ADM and $LSC_{K562/ADM}$ cells. (A) K562 cells and K562/ADM cells were treated with indicated concentrations of PTL for 24h and 48h. The percentage of survival was measured using MTT assay. **(B)** LSC_{K562} cells and $LSC_{K562/ADM}$ cells were treated with indicated concentrations of PTL for 24h and 48h; the survival rate of cells was assessed by MTT assay. Significant difference was compared to control (**P* <0.01).

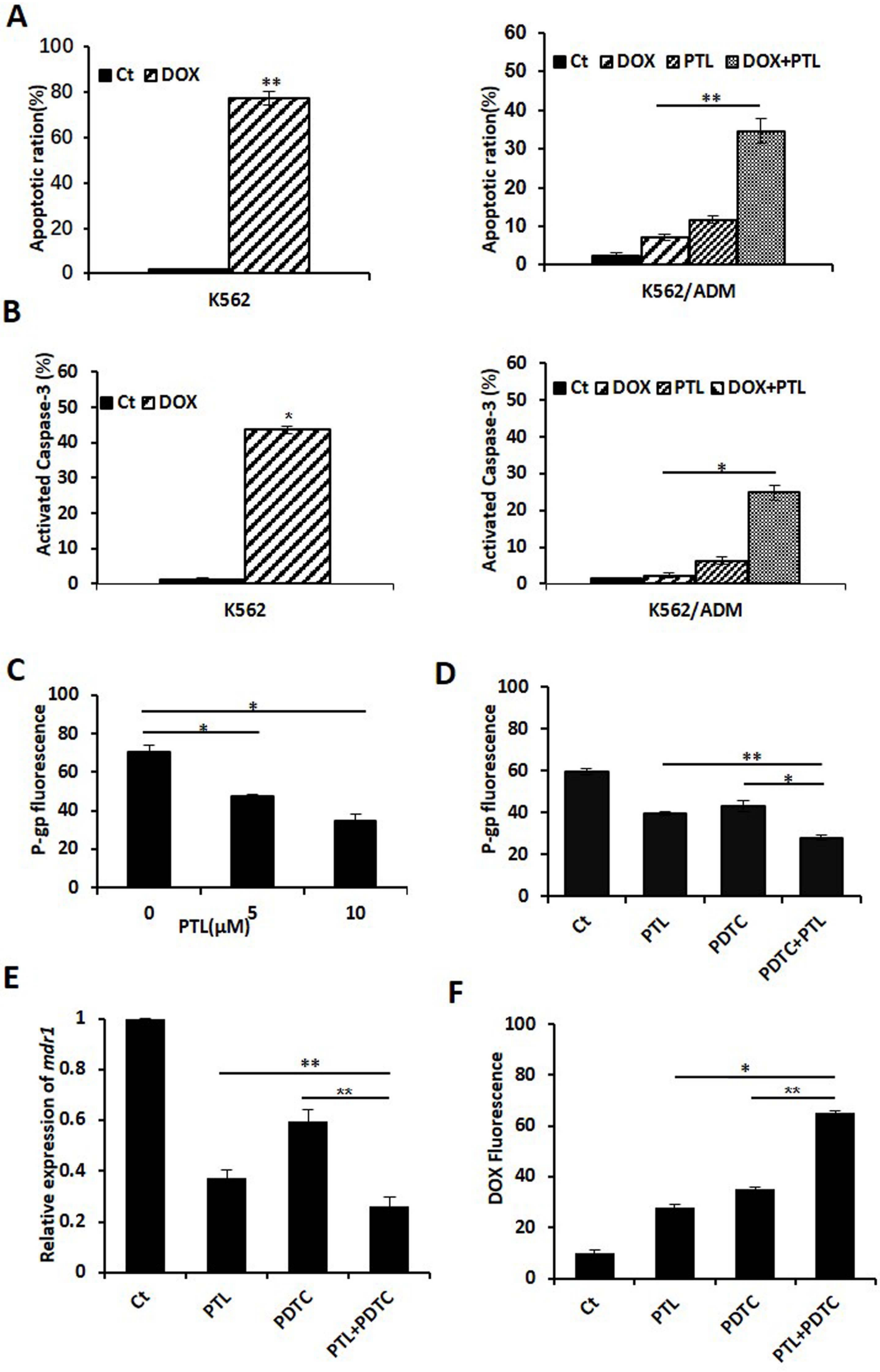

FigureS2. The data of FCM showed that PTL increased susceptibility of K562/ADM cells to DOX through down-regulating expression of P-gp caused by inhibition of NF-κB pathway. (A) After treatment with 2μM DOX for 24h with or without PTL, cells were

labeled with Annexin V/PI and performed analysis of apoptotic rate using FACS. **(B)** cells were treated as described in (A), labeled with activated caspase3 probe, and analyzed positive rates using FACS. **(C)** K562/ADM cells were treated with 10μM PTL for 24h, and P-gp protein level were assessed by FACS. **(D)** K562/ADM cells were treated with 10μM PTL in presence or absence of PDTC for 24h, and P-gp protein level were assessed by FACS. **(E)** K562/ADM cells were treated with 10μM PTL in presence or absence of PDTC for 24h, RT-PCR analysis of *mdr1*. **(F)** K562/ADM cells were treated with 10μM PTL in presence or absence of PDTC for 6h, the fluorescence intensity of DOX were analyzed by FCM. (*$P < 0.01$, **$P < 0.05$).
